# Reductionistic Explanations of Cognitive Information Processing: Bottoming Out in Neurochemistry

**DOI:** 10.3389/fnint.2022.944303

**Published:** 2022-07-04

**Authors:** William Bechtel

**Affiliations:** Department of Philosophy, University of California, San Diego, San Diego, CA, United States

**Keywords:** mechanistic explanation, reduction, control mechanisms, neuropeptides, monoamines

## Abstract

A common motivation for engaging in reductionistic research is to ground explanations in the most basic processes operative in the mechanism responsible for the phenomenon to be explained. I argue for a different motivation—directing inquiry to the level of organization at which the components of a mechanism enable the work that results in the phenomenon. In the context of reductionistic accounts of cognitive information processing I argue that this requires going down to a level that is largely overlooked in these discussions, that of chemistry. In discussions of cognitive information processing, the brain is often viewed as essentially an electrical switching system and many theorists treat electrical switching as the level at which mechanistic explanations should bottom out. I argue, drawing on examples of peptidergic and monoaminergic neurons, that how information is processed is determined by the specific chemical reactions occurring in individual neurons. Accordingly, mechanistic explanations of cognitive information processing need to take into account the chemical reactions involved.

## Introduction

Where should reduction stop? Traditional philosophical accounts of reduction (Nagel, 1961) argue for stopping with the fundamental laws of nature. On these accounts, in a successful reduction, characterizations of higher-level phenomena such as cognitive information processing are derived from these basic laws. New mechanists in philosophy of science challenged the need to invoke laws in explanations in the life sciences and instead argue that explanations often take the form of characterizing the mechanism responsible for the phenomenon being explained (Machamer et al., 2000; Bechtel and Abrahamsen, 2005). These explanations are still reductionistic insofar as they decompose mechanisms into their component entities and activities and appeal to them to explain the phenomenon.^1^ Given the compositional nature of mechanisms, these components can be understood as at a lower level than the mechanism.^2^ While not involving iterated derivations, mechanistic explanations often involve iterated decompositions of entities into components; accordingly, mechanistic explanations can involve multiple descents to lower levels.

On the mechanistic account, the question of where reduction should stop becomes: how many times should one iterate the process of decomposition? Machamer et al. (2000) speak of explanations bottoming out; according to them, the level at which mechanistic explanations bottom out depends on the interests and resources of the investigators. While not denying that explanatory interests are crucial in directing mechanistic inquiry, I argue that there is a principled basis for identifying the level at which mechanistic explanations should bottom out: they should bottom out at the level at which the specific kinds of work that are being performed account for the features of the phenomenon being explained. In the case of cognitive information processing mechanisms, the phenomenon involves the control or regulation of other mechanisms. As I develop in section “Control mechanisms: modifying constraints in controlled mechanisms,” the work that is required to perform control activities involves enabling relevant information to determine the internal constitution of the control mechanism so that it modifies the components of the controlled mechanisms, thereby determining how they operate.

Drawing upon this understanding of control mechanisms, I will argue for a conclusion that will be surprising to many researchers in cognitive science and cognitive and systems neurosciences (It will not, however, be surprising the researchers engaged in cell and molecular research in neuroscience whose research I have drawn on in what follows).^3^ The work that is performed in the nervous system when organisms process information so as to control their activities is not electrical but chemical in nature: it involves the chemical processes through which neurotransmitters (often several) are synthesized in one neuron, released from it, and responded to, often in multiple ways, by other neurons. It is, accordingly, with these various chemical reactions that neuroscientific explanations of cognitive information processing should bottom out.

The importance of the chemical work involved in cognitive information processing is often concealed by a perspective in which synapses are understood on the model of electrical switches. As I will develop in section “The war of the soups and the sparks: who won?,” this perspective has deep roots in the history of neuroscience. Initially many neuroscientists resisted the contention that communication between neurons was chemical, insisting that it was a purely electrical process. Even when the “war of the soups and the sparks” (Valenstein, 2005) ended with the acceptance by the sparks that transmission was chemical, many neuroscientists continued to view neurons as much like electrical switches, with all neurons processing information in essentially the same way. This perspective is reflected in recent work in connectomics and in accounts of artificial neural networks. Connectome maps (Sporns, 2011, 2012, 2015) emphasize structural connections between neurons, and even when they appeal to functional connectivity, they do not address the chemistry through which neurons interact. Brezina (2010), Bargmann (2012), and Nusbaum et al. (2017), among others, have argued for the limitations of connectome maps that fail to take into account the richness of the chemical processes through which neurons interact. In artificial neural network research, the individual nodes are each viewed as summing incoming electrical activity and, based on the sum, initiating a response in the recipient neuron. To explain the processing when neural networks are differently trained, researchers appeal primarily to the weighted connections between neurons (Goodfellow et al., 2016; Aggarwal, 2018). If this reflected how information is processed in our nervous system, neuroscientific explanations could bottom-out with a characterization of how neurons are connected into networks. I will argue, however, that such a model of electrical switching mischaracterizes how the brain processes information. The critical work involved in processing information is performed through the chemical processes through which individual neurons alter their behavior, including their actions on other neurons, in response to specific chemical signals received on their receptors. Critically, these processes are of many different types. These processes provide for a much richer repertoire of ways of processing information than have figured in accounts that construe the brain as processing information through electrical switching.

To a first approximation, the electrical switch model applies to neurons insofar as they communicate through the release at synapses of amino-acid-based neurotransmitters, such as glutamate or GABA, which act on ionotropic receptors (receptors that modify ion channels) in the postsynaptic neuron, altering ion flow across the neuronal membrane and generating a current along it. But this is only one type of transmission between neurons. Even in this case, there are often multiple types of ionotropic receptors that are associated with different channels and produce different postsynaptic currents. Moreover, what current they generate depends not just on the receptor but also current electrical activity and electrochemical gradients in that neuron. I will not develop this, but it further supports the contention that attending to the specific chemical processes through which transmitters are processed in postsynaptic cells is important to understanding neural and cognitive activity. To demonstrate the need to ground cognitive information processing accounts in chemistry I will focus on information processing that involves the release and response to two other types of neurotransmitters, neuropeptides and monoamines, characterizing what is distinctive about the information processing activities in which these transmitters participate.

In section “Information processing with neuropeptides” I focus on information processing relying on peptidergic transmitters. Peptidergic transmitters are employed throughout the brain. One brain region in which they are especially important is the hypothalamus; accordingly, I focus on it as an example. Among other sources and targets, hypothalamic neurons receive inputs from and send outputs to the endocrine system and can be viewed as an extension of it. Unlike amino-acid-based neurotransmitters, neuropeptides are not restricted to the synapse but, like hormones, are disseminated widely and are responded to by whichever cells have appropriate receptors. In most cases, these receptors are metabotropic—they initiate a wide variety of metabolic activities, including gene expression, in the recipient neuron. As a result, the signal is not just an activator or inhibitor of electrical signaling in the recipient cell—what information is processed depends on the peptide synthesized, the receptors that respond to it, the chemical state of the postsynaptic neurons, and the metabolic activities initiated in response. This provides a much richer range of information processing activities than envisaged with the electric switch model.

In section “Information processing with monoamines” I turn to another group of neurotransmitters, monoamines such as dopamine and serotonin. These transmitters are synthesized only in neurons in a limited set of nuclei but are distributed very widely in the brain. In invertebrate research, they were characterized as *neuromodulators* as they were shown to modify how information is processed in local circuits whose pattern of connectivity was not altered. This demonstrated that connectivity alone does not determine how a circuit processes information; it depends on which modulators are bathing the circuit. Insofar as they are released in response to global information and determine the processing in circuits to which they project, they can be viewed as setting the agendas for information processing at classically characterized synapses.

In sections “Information processing with neuropeptides” and “Information processing with monoamines” I will, for the most part, focus on the action of individual neuropeptides and monoamines, but that itself is a serious oversimplification. Nearly fifty years ago some neuroscientists drew attention to the fact that some neurons release multiple transmitters (Burnstock, 1980). Co-transmission is now recognized as the rule, not the exception (Burnstock, 2004; van den Pol and Anthony, 2012; Nusbaum et al., 2017; Svensson et al., 2019). Drawing upon investigations of the feeding circuit in *Aplysia*, Brezina (2010) has shown that the interactions of multiple transmitters are often non-linear. As a result, when released together two or more transmitters may produce an effect that none of them alone produces. Even without developing these complications, the description of the information processing activities involving neuropeptides and monoamines presented in sections “Information processing with neuropeptides” and “Information processing with monoamines” reveals that the brain employs a wide variety of different modes of information processing. It is not limited to or even well characterized in terms of the activities exhibited by electrical switches. Accordingly, as I further develop in the final section, chemical processing between neurons is the appropriate level to bottom out reductionist accounts of the mechanisms of neural information processing.

## Control Mechanisms: Modifying Constraints in Controlled Mechanisms

The standard accounts of mechanisms advanced by the new mechanists in philosophy of science (Machamer et al., 2000; Bechtel and Abrahamsen, 2005) characterize them in terms of their parts, operations, and how these are organized inside mechanisms, not how mechanisms are controlled by external processes. Such control, however, is required if the mechanisms responsible for the core activities of an organism (e.g., contraction of muscles, secretion from glands, synthesis and repair of bodies parts) are to carry out this work^4^ when and only when those phenomena are needed. If these mechanisms are allowed to generate their phenomena (e.g., a muscle is allowed to contract) whenever resources are available, the result is, at best, wasted resources and, worse, generation of phenomena in circumstances in which they are actually harmful to the organism. What cognitive information processing mechanisms do is control other mechanisms.^5^ They do this by performing work on the components of these other mechanisms so that they operate as appropriate on different occasions. Just as with other mechanisms, the work control mechanisms perform depends on their own internal constitution. In virtue of this constitution, they process information that is procured either directly through the making of measurements or from other control mechanisms. In either case, the internal constitution of the control mechanism is altered, resulting in it acquiring information (it is literally, in-formed), which it then processes through the operations its parts perform.^6^

On this framing, mechanistic explanation of a given phenomenon should bottom out with the various work activities that together result in the phenomenon for which an explanation is sought. In the case of muscle contraction, it is the level at which myosin binds to an actin filament and, by hydrolyzing ATP, produces a ofrce that pulls the actin filament along it. In the case of control mechanisms, this is the level at which they are altered by information and, based on that, act on and modify other mechanisms. In the case of muscle, control is achieved through chemical reactions which allow an influx of Ca^2+^ into the cytoplasm of the muscle cell, which serves to expose the binding sites at which myosin can bind actin (Bechtel, 2022). For both control and controlled mechanisms, explanation bottoms out in the characterization of the work that is done to produce the phenomenon.

In some cases, control can be carried out by a single control mechanism. But control can also be spread over multiple mechanisms as long as a signal is passed between them so that the action of the downstream mechanism is dependent on the processing of the upstream mechanisms and ultimately on the ones acquiring the information through making measurements. Such signaling radically expands the potential for information processing. A given control mechanism can be informed by measurements made by multiple mechanisms, process that information in a distinctive way, and send signals to different downstream control mechanisms that carry out further processing or act on controlled mechanisms. There need not be just one pathway through multiple control mechanisms; control mechanisms can form networks. This is exemplified by the integration of neurons into a nervous system in which information procured by some neurons is processed by numerous other neurons and those neurons that directly control muscle cells or secretory cells respond to inputs from many other neurons. The key point remains: Individual acts of information processing are carried out by processes within individual neurons that, in response to inputs, constrain the flow of free energy into the performance of work.

## The War of the Soups and the Sparks: Who Won?

As I indicated in section “Introduction,” the richness of how neurons process information is concealed in the conception of the brain as an electrical switching system. This focus on the nervous system as an electrical system has deep historical roots. Galvani (1791) not only showed that muscles respond to electrical stimulation, but inferred that muscles and nerves, like Leyden jars, contained their own source of electricity. Continuing this line of inquiry, du Bois-Reymond (1848-1884) both provided careful experimental demonstrations of currents in nerves and muscles and identified what he termed “the negative variation” through which nerves transmit signals when stimulated. His student, Bernstein (1868) established that the negative variation, which was later designated as the *action potential*, constituted the nerve pulse. Toward the end of his career Bernstein (1902) showed that, rather than a current, when not stimulated, nerves and muscles exhibit a potential due to ions being unequally distributed across the membrane of the neuron. (For further discussion of this history, see Lenoir, 1987; Bechtel and Vagnino, 2022.)

Once Sherrington (in his contribution to Foster’s 1897, p. 929), named and characterized the synapse, the question arose as to how electrical transmission along one neuron could elicit a response in a post-synaptic neuron. Although Elliott (1904, 1905), Langley (1905), and Dixon (1907) all advanced evidence of chemical transmission, none of them pressed their claims and few researchers at the time accepted that transmission between neurons or neurons and muscles was chemical. Dale (1914), the researcher whose own detailed research on the effects of acetylcholine administration positioned him to embrace chemical transmission, did not [largely due to the lack of any “evidence that a substance resembling acetyl-choline exists in the body at all” (p. 188)]. By the time Dale and Dudley (1929) found acetylcholine as well as histamine in ox and horse spleens, Loewi (1921) had conducted an experiment (conceptually similar to one Dixon had conducted previously) that demonstrated that something he called *Vagusstuff* could be extracted from one heart muscle whose contractions were depressed and administered to another, depressing its contractions. Even when Loewi and Dale were awarded the Nobel Prize in 1936 for chemical transmission at the periphery of the autonomic nervous system, the dominant view was that in the brain and in peripheral nerves controlling skeletal muscles transmission must be electrical. Chemical mediation was deemed to be much too slow to account either for control of skeletal muscles or central processing—the electrical charge was simply understood to jump the gap between neurons. (For indepth historical discussion, see Davenport, 1991; Valenstein, 2005.)

This conflict, which Valenstein (2005) describes as the war between the soups and the sparks, only ceased after Eccles, who had been a chief proponent of electrical transmission, found evidence about inhibitory stimulation that he could not account for with a purely electrical hypothesis (Brock et al., 1952). This resulted in the general acceptance that transmission between neurons involves a chemical process. The issue of the slowness of chemical transmission was partly resolved by the discovery of fast chemical responses. Dale had identified both a fast and slow response to acetylcholine and much of the focus was on the fast response. It took a surprisingly long period to identify the amino acid derivatives glutamate and γ-aminobutyric acid (GABA) as the principal fast-acting neurotransmitters, in part because their presence in the brain was largely attributed to their potential role in metabolism. By the 1970’s they were regarded as “putative neurotransmitters” (Krnjević, 1970; Curtis and Johnston, 1974) and shortly after that glutamate was recognized as the principal excitatory transmitter and GABA as the chief inhibitory transmitter in the mammalian central nervous system.

Referring to a transmitter as excitatory or inhibitory is an oversimplification. Whether in a given case a transmitter generates excitation or inhibition depends on the receptor and conditions in the postsynaptic neuron. In prototypical cases, glutamate and GABA act on ionotropic receptors, opening or closing an ion channel, thereby determining whether an ion (of, e.g., sodium, potassium, calcium, or chloride) is transported through the membrane. This results in either reduced or increased polarization of the membrane and initiates a current along the dendritic membrane. The postsynaptic neuron collects currents generated along its dendritic tree and, if these exceeded a threshold, initiates an action potential along its dendrite. Focusing on this role, chemical processing at synapses can be viewed as simply enabling conduction and switching of electrical signals, rendering the victory of the soups pyrrhic. Attention to the chemical processes may seem to add little to the understanding of neural information processing.

But this is a serious oversimplification. Even if one limits one’s focus to actions on ionotropic receptors, the same transmitter can generate different currents in postsynaptic neurons depending on which ionotropic receptors are present and on the electrochemical gradient across the membrane of the post-synaptic neuron. In addition, though, amino acid transmitters such as glutamate and GABA often bind to not just on ionotropic receptors but also metabotropic receptors, through which they alter metabolic processes, including gene expression, in the postsynaptic cell.

An indication that the electrical transmission account is seriously incomplete is that, once the search for neurotransmitters began, the number of known neurotransmitters mushroomed (there are now more than twenty small molecule neurotransmitters and over a hundred peptidergic transmitters known to be operative in mammalian brains). If all neurotransmitters did were initiate movement of ions across the post-synaptic membrane, one might wonder why nature is so profligate with transmitters?^7^ An alternative perspective that makes sense of the diversity of chemicals acting as neurotransmitters is that the chemical interactions between neurons are not just transmitting information but, depending on the response elicited in the recipient neuron, processing it in different ways. Different receptors for different neurotransmitters result in the recipient neuron behaving differently. To illustrate the implications of focusing on the range of chemical interactions between neurons, I turn in the next two sections to two classes of transmitters that act principally on metabotropic receptors—neuropeptides and monoamines. Once we recognize the diversity of processing provided by chemical transmission between neurons, we can recognize the profound implications of the soups’ victory: it is through a wide range of chemical responses to neural transmissions that information is processed in the mind-brain.

## Information Processing With Neuropeptides

I begin with one of the last class of chemicals to be recognized as neurotransmitters, neuropeptides. In his review of chemicals involved in synaptic transmission, under the category “some other putative transmitters,” (Krnjević, 1974, p. 491) briefly discusses substance P and then, even more briefly, notes that polypeptides had been shown to excite neural activity. He comments, “Whether these are of significance for synaptic function remains to be established.” Substance P had been identified by von Euler and Gaddum (1931) after they found that an extract from whole equine brain depressed blood pressure even after they applied atropine, which was known to inhibit acetylcholine. They viewed it as a second transmitter in their preparation in addition to acetylcholine. Numerous other neuropeptides, such as vasopressin and oxytocin, were discovered in the early 20*^th^* century, but they were at first characterized as hormones and not as neurotransmitters.

Physiologists were investigating hormone signaling even as the sparks dominated discussions of neurotransmission. Drawing on his discovery of secretin, a peptide secreted by the intestines that initiates secretion of digestive fluids in the pancreas (Bayliss and Starling, 1902), Starling (1905) coined the term hormone (from the Greek òρμάω, I excite or arouse) for “chemical messengers which, speeding from cell to cell along the blood stream,… coordinate the activities and growth of different parts of the body.” Subsequent research has resulted in identification of many peptides acting as hormones in coordinating the operation of the various organs responsible for physiological activities such as digestion, respiration, growth, reproduction, and sleep.^8^

Hormone signaling exhibits both features of control mechanisms identified in section “Control Mechanisms: Modifying Constraints in Controlled Mechanisms.” In synthesizing and secreting hormones, cells are responding to measurements of conditions registered in the release of the hormones (e.g., the presence of food). The cells that respond to hormones do so by altering their metabolic processes—catalyzing different reactions or expressing different genes. The differentiation of the processes of generating a signal and responding to it allows for the signal to be distributed to many responders that can respond differently and for responders to respond to different combinations of signals. The evolution of peptidergic neurons can be viewed as an extension of the information processing achieved with hormones.^9^ Essentially, a peptidergic neuron inserts an elongation between a receptor that responds to one or more peptides (and other transmitters) and the machinery for synthesizing new peptides and preparing them for secretion. In this elongation the signal can be propagated either by diffusion through the cytoplasm or electrical transmission along the membrane.

How information is processed by peptidergic neurons depends both on the process by which the peptide is disseminated and on the chemical responses to the peptide. Whereas amino acid transmitters are typically released at the synapse cleft and are restricted to that site, peptidergic transmitters are often volume transmitters—they may be released at various locations on one neuron (van den Pol and Anthony, 2012) and allowed to diffuse through the extracellular matrix as a volume transmitter to wherever they encounter an appropriate receptor, with the physical features of the matrix determining how much and where it diffuses (Agnati et al., 2010; van den Pol and Anthony, 2012; Fuxe et al., 2013). Volume transmitters can engage in multiple interactions—a peptide released by one neuron may act on transmitters of many neurons (this is referred to as *divergence*) and a given neuron may have receptors for transmitters released by many different neurons (*convergence*) (Brezina and Weiss, 1997; Swensen and Marder, 2000; Brezina, 2010). This potential for complexity is further extended when it is recognized that peptidergic neurons often release multiple different peptides as well as other neurotransmitters (Hökfelt et al., 1980; Hökfelt, 2009; Svensson et al., 2019).

**FIGURE 1:**
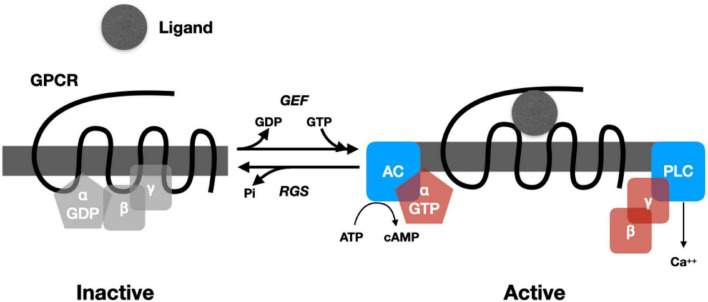
Cartoon representing the operation of a prototypical G-Protein Coupled Receptor. When no ligand is present, G_α_ binds a GDP and the G_βγ_ subunits. When ligand is bound, a GEF (guanine nucleotide exchange factor) promotes exchange of GTP for GDP, causing the subunits of the G-protein to dissociate. G_α_ binds to adenyl cyclase (AC), which then catalyzes cAMP from ATP. G_βγ_ binds phospholipase C, increasing Ca^2+^. Regulator of G-protein signaling (RGS) proteins regulate the hydrolysis of GTP, which returns the G-protein to an inactive state.

In most cases, the response to neuropeptides begins with binding to a G-protein coupled receptor (GPRC) that crosses (seven times) the membrane (van den Pol and Anthony, 2012).^10^ Binding a peptide (or other ligand) on the outside of the cell alters the conformation of the protein, promoting reactions inside the cell. In particular, it activates a guanine nucleotide exchange factor (GEF) that causes the replacement of a GDP by a GTP in a heterotrimeric G-protein complex bound to the receptor on one of its passes inside the cell. The G-protein complex contains two subunits: G_α_ and Gβγ. The Gα subunit is a GTPase that binds and eventually hydrolyzes the GTP. When GEF promotes the exchange of GTP for GDP in the G_α_ subunit (Figure 1), the subunits of the G-protein split apart, allowing each to catalyze reactions. This process is brought to a halt once the G_α_ subunit hydrolyzes GTP to GDP, enabling it to bind to a G_βγ_ subunit and becoming inactive. Regulator of G-protein signaling (RGS) proteins, in turn, modulate the rate of hydrolysis (for a detailed review, see McCudden et al., 2005).

The splitting and activation of the subunits of the G protein can initiate a wide range of biochemical processes in the cell (for an accessible overview, see Marks et al., 2017). The subunits of G-proteins can activate enzymes such as adenylyl cyclase, which generates cyclic AMP (cAMP) from ATP, and phospholipase C, which, via the synthesis of inositol trisphosphate, generates an increase in Ca^2+^. Both cAMP and Ca^2+^ are intracellular signals (second messengers) that initiate subsequent reactions depending on the constituents of the cell (cAMP through whatever protein kinase A is available and Ca^2+^ through whatever protein kinase C is available). These diverse chemical reactions constitute the processing of the signal. Among the results of these reactions is altered gene expression, including the synthesis of new peptides. In addition to being synthesized in the endoplasmic reticulum, new peptides are subject to extensive post-translational modifications in the Golgi apparatus (different modifications resulting in different peptides) and then packaging into large dense core vesicles. One of the functions of a small but relatively long-lasting increase in Ca^2+^ concentration in the cytoplasm is the release of the contents of these vesicles into the extracellular matrix.

Neurons responding to and releasing neuropeptides play an especially important role in the hypothalamus. The hypothalamus consists of highly interconnected nuclei, each typically containing multiple cell types distinguished by their receptors and their machinery for synthesizing new peptides (Leng, 2018). Many of these nuclei are located adjacent to the median eminence at the base of the diencephalon, an ideal location for extending endocrine signaling since there is no blood-brain barrier at the median eminence. Instead, the fenestrated capillaries allow hormones to act on neurons in the hypothalamus and for peptides synthesized by hypothalamic neurons either to act as hormones by entering the bloodstream directly (oxytocin and vasopressin) or to initiate the synthesis of hormones in the pituitary. To illustrate the variety of information processing activities of peptidergic neurons in the hypothalamus, I will briefly describe the function of two peptides—orexin and vasopressin—released by populations of hypothalamic neurons.

When orexin-releasing neurons were discovered in the lateral hypothalamic area in the late 1990’s (de Lecea et al., 1998; Sakurai et al., 1998), they were interpreted as promoting feeding behavior (the name *orexin* is derived from the Greek word for appetite). Researchers soon demonstrated that these neurons integrate signals from two populations of neurons in another hypothalamic nucleus, the arcuate nucleus. Neurons in one arcuate population respond to peptides such as leptin, which is released from adipose cells in proportion to fat mass, and release proopiomelanocortin (POMC). POMC can be viewed as signaling satiety (Yeo et al., 2021). Neurons in the other population respond to peptides such as ghrelin, which is synthesized in the gut and duodenum when no food is present. It generates neuropeptide Y and agouti-related peptide; high concentration of these peptides can be viewed as signaling hunger (Aponte et al., 2011; Al Massadi et al., 2017). (Although leptin and ghrelin are the best studied of these peptides, each population of arcuate nucleus neurons receives multiple peptidergic signals and integrates these to arrive at its input to the orexin neurons.) Orexin neurons have receptors for POMC, neuropeptide Y, and agouti-related peptide; these differentially effect their synthesis of orexin. Through their projections these neurons release orexin broadly through the brain; in many locations the presence of orexin initiates feeding behavior. Orexin-releasing neurons can thus be viewed as assessing information about the organism’s nutritional state and reaching a decision as to whether to initiate feeding behavior. But the story is much more complex. Shortly after they were discovered, orexin neurons were also found to be especially active during sleep-to-wake transitions. Stimulation of orexin neurons was found to promote waking.^11^ Fittingly, these neurons also receive inputs from the reticular activating system in the brain stem. This revealed that orexin-releasing neurons integrate nutritional information and a variety of activating signals, initiating responses based on multiple sources of information. Subsequent research revealed that orexin-releasing neurons respond to an even wider range of peptides, signaling a variety of cell states, and contribute to initiating a broad range of cell responses (for a review, see Arrigoni et al., 2019).

Vasopressin-synthesizing neurons in the supraoptic and paraventricular nuclei of the hypothalamus reveal a similar pattern of integrating multiple sources of information and generating multiple responses (Stoop, 2012; Sternson, 2013; Leng, 2018; Watts et al., 2021). Neurons in these two nuclei receive excitatory inputs from the amygdala and inhibitory inputs from the hippocampus as well as noradrenergic and monoaminergic inputs from the brainstem. One result is that they register osmolarity and low-blood volume, as well as various stressors. Different cell populations in these nuclei synthesize and release vasopressin to different locations (Watts, 2010). Magnocellular neurons project into the posterior pituitary where they release vasopressin into the blood stream (Nestler et al., 2015, chapter 10). Vasopressin released into the blood has different downstream effects depending on which cells have either V1 or V2 receptors (each initiates a distinct metabolic cascade). (In addition to vasopressin, these neurons also release the opioid peptides enkephalin and dynorphin, neuropeptide Y, cholecystokinin, and galanin, each of which acts on cells with appropriate receptors.) For example, the V2 receptor on the distal nephron of the kidney initiates the synthesis and insertion of water channels that result in the reabsorption of water into the circulation (in the process, rendering the urine more concentrated). Vasopressin in the blood also acts on V1a receptors in arterioles, causing them to contract and thereby raise blood pressure.

A second population of parvocellular neurons releases vasopressin, together with corticotropin releasing factor (CRF), into to the hypophyseal–portal circulation, which drains into the anterior pituitary (Nestler et al., 2015, chapter 10). There vasopressin and CRF together bind cells with V1b receptors and initiate a sequence of reactions beginning with the synthesis of POMC (discussed above as signaling satiety), which in turn simulates synthesis and release of ACTH (Aguilera et al., 2008). ACTH both feeds back to inhibit POMC transcription and acts on receptors on different populations of cells in the adrenal cortex to initiate the synthesis and secretion from cholesterol of either glucocorticoids (cortisol) or other steroids including aldosterone and adrenal androgens. These in turn recruit energy for a flight-or-flight response. (Glucocorticoids also feeds back to repress both CRF and ACTH synthesis, thus stopping the action initiated by vasopressin.) In addition to these actions on the endocrine system, vasopressin synthesizing neurons also release vasopressin into other brain regions where it is implicated in reducing aggression and affiliative behaviors.

The two example neuropeptides I have discussed, orexin and vasopressin, reveal important features of how peptidergic neurons process information in the brain. The neurons that synthesize them do so in response to appropriate ligands (each of which carries information about one or more conditions in the organism). These ligands bind to GPRCs that, depending on the particular type of cell in which they occur, trigger the synthesis of specific peptides that can then be released into the extracellular matrix. The near endless variety of possible peptides, receptors, and intracellular signaling pathways (Brezina, 2010) allows peptidergic neurons to process information in a vast number of ways. Moreover, the ability of these neurons to incorporate receptors for multiple inputs and generate multiple outputs allows them to integrate information. In this way, they build upon the information processing capacities of the endocrine system, extending its capacity to process information. To understand how these neurons process information, one needs to take into account the different peptides, receptors, and intracellular signaling pathways involved, including the frequent release of multiple peptides by the same neuron and response to multiple peptides by a single neuron.

## Information Processing With Monoamines

To further develop the theme that one needs to ground accounts of neural information processing in the chemical activities in neurons, I turn to a second class of neurotransmitters, the monoamines norepinephrine, dopamine, and serotonin. They were among the earliest chemicals identified as neurotransmitters when they were found to meet the criteria of occurring naturally in brains and when administered, eliciting a detectable response. Only as researchers were able to localize their synthesis in the brain and investigate the receptors that responded to them did they come to recognize the distinctive type of information processing they support. On the one hand, while they are disseminated widely, in vertebrates they are only synthesized in a select set of nuclei—norepinephrine in the locus coeruleus and other nuclei in the pons and medulla, dopamine in the substantia nigra pars compactus (SNc) and the ventral tegmental area (VTA),^12^ and serotonin in the raphe nuclei. Like neuropeptides, they mostly act through GPCRs. What is distinctive is how they act on other neurons—they alter the responses of recipient neurons to the main excitatory or inhibitory transmitters (glutamate and GABA). Accordingly, they are often referred to as neuromodulators.^13^ That, however, understates their role in determining how information is processed through these more traditional synapses. As they configure how circuits respond to electrical signals, them might be viewed as setting the information processing agenda for these neural circuits.^14^

The fact that neuromodulators determine how neural circuits process information was first and most clearly demonstrated in invertebrate research. In many invertebrates the identity and connectivity of neurons is consistent organism to organism. This has made it possible to develop species-wide connectomes (maps of neural connectivity). Through serial electron microscopy, White et al. (1986) created a nearly complete map of neurons and their connections in the hermaphrodite nematode *C. elegans*. Based on this, researchers began to develop accounts of how specific circuits process information (Chalfie et al., 1985). However, other researchers discovered that the responses of neurons in these circuits can be modified by application of neuromodulators such as dopamine. Bargmann (2012) describes numerous cases in *C. elegans* in which application of neuromodulators changes the response properties of specific circuits without changing their physical connections. Marder has provided similar examples in a specific circuit, the stomatogastric ganglion network, in the lobster. This network of about 27 neurons regulates the foregut muscles that grind food and force it down to the gut. The network can be extracted and studied *in vitro*. Such investigations revealed that the circuit consists of two central pattern generators, one of which, the pyloric network, is constantly rhythmic while the other, the gastric mill network, generates rhythms only when it receives modulatory inputs produced by sensory inputs. Although there is a fixed pattern of physical connections between these neurons, the circuits exhibit different behavior when different monoamines and other neurotransmitters are added to the preparation (Marder and Bucher, 2007). The effects of dopamine are particularly dramatic as each neuron has dopamine receptors but responds differently to the addition of dopamine.^15^ The ability of neuromodulators to alter circuit behavior in invertebrates turns out to be the rule, not the exception (Marder, 2012).

Although it is more difficult to study the effects of monoamines on specific circuits in vertebrates, their effects in modulating neural activities are clear. The effects of dopamine on processing in the basal ganglia are illustrative. The basal ganglia are a network of nuclei implicated in selecting which other neural circuits process information. By default, the output regions of the basal ganglia send inhibitory GABAergic projections to regions throughout the brain (both those involved directly in action and those involved in central information processing). Only when activity in the basal ganglia inhibits these inhibitory outputs can these other brain regions carry out their activities. The basal ganglia are connected in loops to these other areas. In each loop, a brain region sends excitatory glutamatergic inputs to two sets of medium spiny neurons (MSNs) in the striatum, the input region of the basal ganglia, and the output regions of the basal ganglia act via the return loop either to maintain the inhibition or release it.

The standard account of the operation of the basal ganglia function (originally advanced by Albin et al., 1989, to account for the features of different disorders associated with the basal ganglia) identifies two pathways originating with the MSNs in the striatum that receive the inputs. In what is referred to as the direct pathway, MSNs with D1 receptors send inhibitory projections directly to the output nuclei of the basal ganglia. By inhibiting these inhibitory outputs, active neurons with D1 receptors release the connected region from inhibition, allowing it to process information. Neurons with D2 receptors, in contrast, send inhibitory projects to intermediate regions of the basal ganglia, inhibiting their inhibitory effects on the output regions; the net effect is to enhance their inhibitory action on other brain regions. For contemporary presentations of this account, see Gerfen and Bolam (2016) and Clark et al. (2018). In developing a computational model of decision making based on this account, Hazy et al. (2007) characterize the direct pathway as generating a Go signal while the indirect pathway generates a NoGo signal.

On the standard account, it is the effect of dopamine in binding to the D1 and D2 receptors that determines the output of the basal ganglia.^16^ When dopamine binds the D1 receptor, it initiates a cascade involving cAMP and protein a kinase A (PKA), with the PKA initiating responses that enhance the expression of both alpha-amino-3-hydroxy-5-methyl-4-isoxazolepropionate receptors (AMPARs) and N methyl-D-aspartate receptors (NMDARs), ionotropic receptors that respond to glutamate. The effect is to increase the responsiveness of these neurons to sustained glutamate inputs but to decrease it to transient inputs (Shen et al., 2016). When dopamine binds the DR2 receptor, it initiates responses including removal of AMPARs from the cell membrane and changes in Ca^2+^ and Na^+^ ion channels. In addition to these immediate effects, the combined action of dopamine and brain-derived neurotropic factor released by cortical inputs on D1 receptors acts on a tyrosine receptor kinase B. This serves to initiate long-term activation (LTP), enhancing the likelihood that the neuron will respond to the same glutamatergic input in the future. In neurons with D2 receptors, when glutamate bind the mGluR5 receptor while Ca^2+^ is released into the neuron, it initiates long-term depression (LTD) (Shen et al., 2016).

Varying the amount of dopamine reaching the striatum from the SNc can thus alter how the basal ganglia processes inputs both immediately and in the longer term. When it is drastically reduced, as in Parkinson’s, the response to inputs to D1 neurons is reduced while that to D2 neurons is enhanced, thereby reducing activity along the direct pathway and the release of other brain areas from inhibition. This explains the inability of Parkinson’s patients to initiate voluntary actions. In most individuals, dopamine modulates how the basal ganglia process information. Starting with Schultz (Schultz et al., 1997; Schultz, 1998), a number of theorists have viewed dopamine as constituting a reward signal and have interpreted it as key to implementing reinforcement learning (as developed by AI theorists Sutton and Barto, 2018) by enabling neurons in the striatum to compare expected with actual reward and use that as a basis of learning. Others, such as Redgrave et al. (2016), contend instead that dopamine signaling enables stiratal neurons to assess whether the organism is the agent of an outcome by detecting unexpected outcomes and relating them to efferent copies of motor commands. While there is disagreement of how to interpret the effects of dopamine on striatal neurons, all admit it plays a major role in structuring processing in the striatum and downstream in the basal ganglia.

**FIGURE 2:**
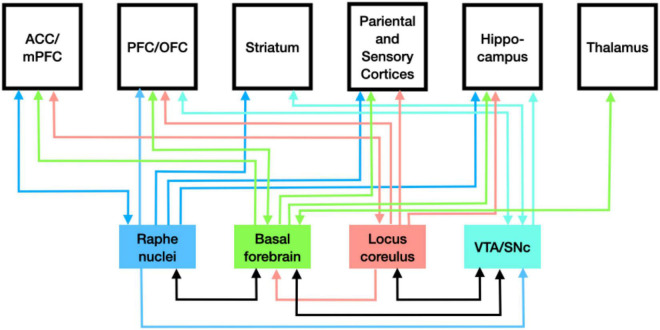
Pattern of distribution of monoamines from the loci where each is synthesized.

The action of dopamine produced in the SNc on the striatum of the basal ganglia is just one instance of neuromodulatory activity of the monoamines. As noted above, in vertebrates, each of the monoamines is synthesized only in select nuclei but neurons in these nuclei extend axons widely through the brain. These neurons release the monoamines as volume transmitters into the extracellular matrix where they able to bind a variety of receptor types on different neurons in the region and alter how they respond to amino acid transmitters at synapses. When the whole set of monoamines is considered, they can be seen to have multiple effects on how other brain regions process information. As indicated in Figure 2, the pattern of distribution is complicated. Each nucleus in which monoamines are synthesized sends projections to many areas, including those where other monoamines are synthesized. Brain areas often receive inputs from multiple monoamines. Moreover, in many cases the projections are recurrent. The broad distribution from select nuclei suggests that these transmitters can determine how the whole brain processes information. This is supported by the range of neurological and psychiatric disorders associated with disrupted monoamine response in the various brain regions. Researchers face considerable challenges in determining how the monoamines individually and collectively modulate neural information processing, but it is apparent that they play different roles than just inputs to electrical switches.

## Conclusion: Grounding Information Processing Explanations in Chemical Activity

Electrical switching has long been the model of how neurons process information. On such a perspective, it would seem sufficient for mechanistic explanations of cognitive information processing to bottom out with the connectivity of neurons. There would seem to be little reason to decompose further to the chemical processes of synthesizing and responding to neurotransmitters. One could acknowledge, as the sparks did in ending their war with the soups, that chemical transmitters intervened between neurons, but still insist that the details of chemical activity would not further enlighten our understanding of how information is processed in the brain. Connectomic analyses and neural network models based on them could explain how information is processed.

The variety of neurotransmitters and the diversity of ways neurons respond to them suggests that, on the contrary, considerations of the chemical processes are pertinent to understanding how nervous systems process information. Neuropeptides and monoamines figure in quite different information processing than amino-acid-based transmitters acting on ionotropic receptors. The response to neuropeptides is not just the generation of an action potential in the recipient cell but a wide range of metabolic activities, including the synthesis of new peptides. The response to monoamines can significantly alter the processing in a circuit by amino-acid-based transmitters. The details of the chemical processing between neurons matters for how information is processed. As a foundation for understanding how brains process information, researchers need, in addition to a connectome detailing synaptic connections, a chemoconnectome: “an entire set of neurotransmitters, neuromodulators, neuropeptides, and receptors supporting chemical transmission in an animal (Moroz, 2021).

I have approached the issue of reduction from the perspective of developing mechanistic explanations of control mechanisms. A central feature of mechanistic approaches is decomposing systems. Until one reaches true atoms (indivisible components), further decomposition is always possible. Accordingly, one could argue that while chemical processes are important, mechanistic explanations of neural information processing should not bottom out there but, for example, continue on to the quantum processes at work in these chemical reactions. The account of mechanistic explanation in terms of the work performed by the mechanism, however, shows why further decomposition is not likely to be informative. In the case of control mechanisms, the relevant work is processing information. With the chemical processes between neurons, one has reached a level at which one can account for the different ways in which information is processed. Further decomposition will not provide additional illumination about the ways information is processed in the brain.

In advocating for explanations of neural information processing bottoming out in chemical processing, I am not arguing that only the chemical level is required to understand such information processing. Organization at multiple levels helps determine how information is directed through an organism. Patterns of connections between neurons is important, as is the organization of neurons into nuclei and brain structures. Even higher levels of organization are also relevant, including levels that integrate neurons with different organs and connect activities in organisms to entities in their environment, including the social environment. In requiring both reductionistic and holistic research, mechanistic reduction differs from Bickle’s (2003, 2006) characterization of ruthless reduction. Insofar as one explanatory goal is to understand how circuits in the brain process information, though, the level of chemical processes that mediate between neurons is of critical importance as it is a level at which the work of processing information in particular ways is carried out.

## Data Availability Statement

The original contributions presented in the study are included in the article. Further inquiries can be directed to the corresponding author/s.

## Author Contributions

The author confirms being the sole contributor of this work and has approved it for publication.

## Conflict of Interest

The author declares that the research was conducted in the absence of any commercial or financial relationships that could be construed as a potential conflict of interest.

## Publisher’s Note

All claims expressed in this article are solely those of the authors and do not necessarily represent those of their affiliated organizations, or those of the publisher, the editors and the reviewers. Any product that may be evaluated in this article, or claim that may be made by its manufacturer, is not guaranteed or endorsed by the publisher.

## Footnotes

1 They also appeal to how these parts are organized and how the whole mechanism is situated. Different ways of organizing components, and different ways of embedding them in a larger context, can result in producing different phenomena from the same parts. Insofar as they recognize the importance of organization and the situatedness of mechanisms (Bechtel, 2009), mechanistic explanations are not solely reductionistic and do not fit the mode of Bickle’s (2003, 2006) account of ruthless reduction. Nonetheless, appeal to components is a fundamentally reductionistic feature of mechanistic explanations.

2 Mechanistic levels are defined relative to the mechanism that is being decomposed. A mechanism may be decomposed into components of vastly varying sizes, which nonetheless interact to produce the phenomenon and so are then denizens of the same level. Decomposing a different mechanism may combine in one level entities at a different level in another mechanism. Accordingly, as argued by Craver and Bechtel (2007), mechanistic accounts do not assume levels that span the natural world.

3 The balkanization of science is often much lamented and the failure of many cognitive and systems-level neuroscientists to draw upon the results of decades of research on chemical signaling in the brain is another example. Often pursuing one’s research in ignorance of potentially relevant contributions of others allows for important advances. This has certainly been the case in cognitive science and cognitive and systems neuroscience. Yet, as I will argue, what is overlooked is extremely important for the understanding one is trying to develop and has the potential to amend and enrich our understanding of how brains contribute to cognition and control behavior.

4 Traditionally, new mechanists have not characterized mechanisms as performing work. Focusing on work, however, highlights another omission in new mechanist accounts—that mechanisms require free energy to produce phenomena. Winning and Bechtel (2018; see also Bechtel and Bollhagen, 2021), drawing inspiration from Pattee’s (1972a,b) have characterized mechanisms as performing work as a result of the configuration of their components constraining flows of free energy. In biological mechanisms, free energy usually takes the form of ATP. Even without specifically identifying where free energy is released and how the components constrain its effects, one can characterize the work that is thereby accomplished. That is the perspective adopted in this paper.

5 Single cell organisms also require such control mechanisms; for discussion, see Bich and Bechtel (2022).

6 The concept *information* has been characterized in different ways. Shannon (1948) developed a mathematical analysis of the quantity of information carried by a signal in terms of how much it reduced uncertainty. Dretske (1981) advanced a semantic characterization of information that emphasizes its content—what the signal is carrying information about. Dretske offers a basically Humean causal account of how a signal acquires content. An Aristotelian perspective in which a causal process alters the form of an object more fully captures Pattee’s (1972a,b) undertanding in which a control process performs and exectues a process based on a measurement in which a state of the control system comes to correspond to the property being measured.

7 Moroz et al. (2021, p. 7) argue that, on the electrical switching account, two, or even one, transmitter would suffice: “If a chemical messenger acts only as a pure transmitter(= messenger) at the synaptic cleft within a specific wiring diagram, only two neurotransmitters are needed (e.g., for excitation and inhibition, respectively). If there are two different receptors for the same transmitter (to induce excitation and inhibition)—then even one transmitter might be sufficient.” Considering circuit design, there may be good reasons to have more than one or two transmitters (e.g., to keep different messages segregated), but the number that have been found vastly exceeds what would be required to satisfy circuit requirements.

8 Hormone signaling itself serves to process information. In animals without neurons, such as *Trichoplax adhaerens*, which has just six cell types, hormones coordinate activities in different cells and enable a variety of different behaviors (Senatore et al., 2017). Similar signaling occurs within plants and between single-cell organisms, including prokaryotes that act cooperatively in biofilms. Such signaling typically involves the secretion of peptides from one cell that are taken up by and metabolized in other cells. Such signaling, however, is not limited to peptide transmission: Prindle et al. (2015) have shown the bacteria also make use of electrical currents transmitted along cell membranes to coordinate activity in biofilms, even among bacteria of different species.

9 Moroz et al. (2021) have argued that neurons first evolved to extend peptidergic signaling.

10 There has been almost no discussion of GPCRs in the philosophical literature. A notable exception is Barwich and Bschir’s (2017) analysis how GPCRs went from hypothetical posits to a class of entities occurring in nature. They argue that success in manipulating GPCRs played an important role, but only once researchers had “an adequate conceptual grasp of their potential structural and functional roles” (p. 1333). Barwich and Bschir emphasize the historical process of “epistemic iteration” (a concept they barrow from Chang, 2004)—an interplay between conceptual development and experimental interventions.

11 The output from orexin neurons is complex, often involving the corelease of orexin and glutamate, with the two transmitters operating on different timescales (Schöne et al., 2014).

12 A small collection of neurons in the arcuate nucleus of the hypothalamus also synthesizes dopamine. Its release suppresses synthesis of prolactin in the anterior pituitary. Dopamine is also synthesized in local circuit neurons in the retina.

13 The term neuromodulator is used in different ways by different researchers. In an early review of neuromodulators, Kupfermann (1979, p. 448) asserts that they “do not simply excite or inhibit an electrically excitable cell, but rather are involved in altering the effects of other events occurring at the cell.” Katz (1999, p. 3) offers a far more inclusive definition: “Any communication between neurons, caused by release of a chemical, that is either not fast, or not point-to-point, or not simply excitation or inhibition will be classified as neuromodulatory.” Under either definition, the term includes the neuropeptides discussed in the previous section. In this section I single out the monoamines for their distinctive mode of neuromodulation.

14 An important feature of neuromodulators, emphasized by Brezina (2010), is that commonly multiple neuromodulators are acting at the same time, resulting in multiple non-linear interactions that determine the output in any given case.

15 These circuits exhibit yet further complexity as a result of co-transmission of neuropeptides and small molecules (Nusbaum et al., 2017).

16 The standard account is likely to be oversimplified. Graybiel (2005) identified six challenges based on then recent empirical evidence (e.g., evidence that there are many more collateral connections between the supposedly independent pathways, that the outputs of the basal ganglia are not solely inhibitory, and that the D1 and D2 neurons receive different inputs from cortex). Doubts about the standard account do not minimize the importance of dopaminergic signaling to the striatum. In her own research, Graybiel has investigated the role of dopamine projections to the striosomes of the striatum as relating mood and emotion to decision making (Graybiel and Grafton, 2015) and the role of dopamine in the acquisition of habits by modifying the behavior of striatal neurons (Graybiel and Grafton, 2015).
